# The genome sequence of the model ascomycete fungus *Podospora anserina*

**DOI:** 10.1186/gb-2008-9-5-r77

**Published:** 2008-05-06

**Authors:** Eric Espagne, Olivier Lespinet, Fabienne Malagnac, Corinne Da Silva, Olivier Jaillon, Betina M Porcel, Arnaud Couloux, Jean-Marc Aury, Béatrice Ségurens, Julie Poulain, Véronique Anthouard, Sandrine Grossetete, Hamid Khalili, Evelyne Coppin, Michelle Déquard-Chablat, Marguerite Picard, Véronique Contamine, Sylvie Arnaise, Anne Bourdais, Véronique Berteaux-Lecellier, Daniel Gautheret, Ronald P de Vries, Evy Battaglia, Pedro M Coutinho, Etienne GJ Danchin, Bernard Henrissat, Riyad EL Khoury, Annie Sainsard-Chanet, Antoine Boivin, Bérangère Pinan-Lucarré, Carole H Sellem, Robert Debuchy, Patrick Wincker, Jean Weissenbach, Philippe Silar

**Affiliations:** 1 Univ Paris-Sud, Institut de Génétique et Microbiologie, UMR8621, 91405 Orsay cedex, France; 2 CNRS, Institut de Génétique et Microbiologie, UMR8621, 91405 Orsay cedex, France; 3 UFR de Biochimie, Université de Paris 7 - Denis Diderot, case 7006, place Jussieu, 75005, Paris, France; 4 Genoscope (CEA) and UMR 8030 CNRS-Genoscope-Université d'Evry, rue Gaston Crémieux CP5706, 91057 Evry, France; 5 Microbiology, Department of Biology, Utrecht University, Padulaan, 3584 CH Utrecht, The Netherlands; 6 UMR 6098, Architecture et Fonction des Macromolecules Biologiques, CNRS/univ. Aix-Marseille I et II, Marseille, France; 7 CNRS, Centre de Génétique Moléculaire, UPR 2167, 91198 Gif-sur-Yvette, France; 8 Université Paris-Sud, Orsay, 91405, France; 9 Institut de Biochimie et de Génétique Cellulaires, UMR 5095 CNRS/Université de Bordeaux 2, rue Camille St. Saëns, 33077 Bordeaux Cedex, France

## Abstract

**Background:**

The dung-inhabiting ascomycete fungus *Podospora anserina *is a model used to study various aspects of eukaryotic and fungal biology, such as ageing, prions and sexual development.

**Results:**

We present a 10X draft sequence of *P. anserina *genome, linked to the sequences of a large expressed sequence tag collection. Similar to higher eukaryotes, the *P. anserina *transcription/splicing machinery generates numerous non-conventional transcripts. Comparison of the *P. anserina *genome and orthologous gene set with the one of its close relatives, *Neurospora crassa*, shows that synteny is poorly conserved, the main result of evolution being gene shuffling in the same chromosome. The *P. anserina *genome contains fewer repeated sequences and has evolved new genes by duplication since its separation from *N. crassa*, despite the presence of the repeat induced point mutation mechanism that mutates duplicated sequences. We also provide evidence that frequent gene loss took place in the lineages leading to *P. anserina *and *N. crassa*. *P. anserina *contains a large and highly specialized set of genes involved in utilization of natural carbon sources commonly found in its natural biotope. It includes genes potentially involved in lignin degradation and efficient cellulose breakdown.

**Conclusion:**

The features of the *P. anserina *genome indicate a highly dynamic evolution since the divergence of *P. anserina *and *N. crassa*, leading to the ability of the former to use specific complex carbon sources that match its needs in its natural biotope.

## Abstract 2

A 10X draft sequence of *Podospora anserina* genome shows highly dynamic evolution since its divergence from *Neurospora crassa*.

## Background

With one billion years of evolution [1], probably more than one million species [2] and a biomass that may exceed that of animals [3,4], eumycete fungi form one of the most successful groups of eukaryotes. Not surprisingly, they have developed numerous adaptations allowing them to cope with highly diverse environmental conditions. Presently, virtually all biotopes, with the exception of extreme biotopes (that is, hyperthermophilic areas), contain some representative eumycetes. They feed by osmotrophy and import through very efficient transporters the nutrients they take up from the environment, often by degrading complex material, such as plant cell walls, that few other organisms can use.

Eumycete fungi have a huge impact on the global carbon cycle in terrestrial biotopes. Some species associate with plant and algae, helping them to scavenge mineral nutrients and to cope with various stresses, such as poor soils, desiccation, parasites and herbivore damage. These mutualistic relationships lead to better carbon dioxide fixation. In contrast, many species parasitize plants and algae, resulting in reduced carbon fixation [5], as well as causing serious economic losses to human agriculture. The majority, however, are saprobic and live on dead plant material, such as fallen plant debris, plants ingested by herbivores or the remains of plants in feces of herbivores. It is estimated that saprobes release 85 billion tons of carbon dioxide annually [6,7], much higher than the 7 billion tons emitted by humans [8]. Finally, some fungi can infect and kill animals, especially invertebrates, which results in diminished carbon fluxes within the food chain. A few are opportunists able to infect humans. Impact on human health is increasing because of the higher prevalence of immunodeficiency, a condition favoring fungal infection.

In addition to these global effects, eumycetes impact their biotope and humans in many ways. Indeed, humans have been using them for thousands of years as food, to process other plant or animal materials and to produce compounds of medicinal interest. A few species degrade human artifacts, causing permanent damage to irreplaceable items. Furthermore, due to their ease of handling, some species, such as *Saccharomyces cerevisiae *or *Neurospora crassa*, have been exploited as research tools to make fundamental biological discoveries. In recent years, a number of genome initiatives have been launched to further knowledge of the biology and evolution of these organisms. Presently, a large effort is dedicated to saccharomycotina yeasts (formerly hemiascomycetes) [9]. Other efforts are concentrated towards human parasites and plant mutualists or pathogens. The genomes of *Magnaporthe grisea*, a rice pathogen, *Fusarium graminearum*, a wheat pathogen, *Ustilago maydis*, a maize pathogen, *Cryptococcus neorformans *and *Aspergillus fumigatus*, two human pathogens, have been published [10-14]. In addition, saprobic fungi are also considered, since the genome sequences of the basidiomycete *Phanerochaete chrysosporium *[15], of the ascomycetes *N. crassa *[16] and *Schizosaccharomyces pombe *[17], and three strictly saprobic Aspergilli, *A. nidulans*, *A. oryzae *and *A. niger *[18-20], are available.

**Figure 1:**
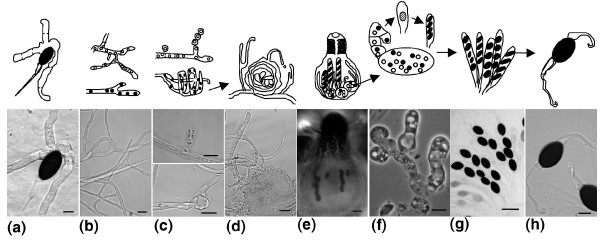
The major stages of the life cycle of *P. anserina *as illustrated by light microphotography, with a corresponding schematic representation shown above. **(a) **The cycle starts with the germination of an ascospore, after the transit in the digestive tract of an herbivore in the wild. **(b) **Then, a mycelium, which usually carries two different and sexually compatible nuclei (pseudo-homothallism), called *mat+ *and *mat-*, develops and invades the substratum. **(c) **On this mycelium, male (top; microconidia) and female (bottom; ascogonium) gametes of both mating types differentiate after three days. In the absence of fertilization, ascogonium can develop into protoperithecium by recruiting hyphae proliferating from nearby cells. **(d) **This structure, in which an envelope protects the ascogonial cell, awaits fertilization. **(e,f) **This occurs only between *mat+ *and *mat- *sexually compatible gametes (heterothallism) and triggers the development completed in four days of a complex fructification (e) or perithecium, in which the dikaryotic mat+/mat- fertilized ascogonium gives rise to dikaryotic ascogenous hyphae (f). **(g) **These eventually undergo meiosis and differentiate into ascii, mostly with four binucleate mat+/mat- ascospores (pseudo-homothallism), but sometime with three large binucleate ascospores and two smaller uninucleate ones (bottom asci is five-spored). Unlike those issued from large binucleate ascospores, mycelia issued from these smaller ascospores are self-sterile because their nuclei carry only one mating type. **(h) **When ripe, ascospores are expelled from perithecia and land on nearby vegetation awaiting ingestion by an herbivore. Scale bar: 10 μm in (a-d,f,h); 200 μm in (e,g).

Because of its ease of culture and the speed of its sexual cycle, which is completed within a week, the saprobic filamentous ascomycete *Podospora anserina *(Figure 1) has long been used as a model fungus in several laboratories [21,22] to study general biological problems, such as ageing, meiosis, prion and related protein-based inheritance, and some topics more restricted to fungi, such as sexual reproduction, heterokaryon formation and hyphal interference (Table 1). *P. anserina *and *N. crassa *both belong to the sordariomycete clade of the pezizomycotina (formerly euascomycete). Based on the sequence divergence between the *P. anserina *and *N. crassa *18S rRNA, the split between the two species has been estimated to have occurred at least 75 million years ago [23]. However, the average amino acid identity between orthologous proteins of the two species is 60-70% [24], the same percentage observed between human and teleost fishes [25], which diverged about 450 million years ago [26,27]. It is not surprising, therefore, that despite similar life cycles and saprobic lifestyles, each species has adopted a particular biotope and displays many specific features (Table 2). To better comprehend the gene repertoire enabling *P. anserina *to adapt to its biotope and permit this fungus to efficiently complete its life cycle, we have undertaken to determine the genome sequence of *P. anserina *and have compared it to that of *N. crassa*, its closest relative for which the genome sequence is already known. We started with a pilot project of about 500 kb (about 1.5% of the genome) [24] and in this paper we present the establishment of a 10X draft sequence.

**Table 1:** Areas of research that should benefit from the *P. anserina *complete genome sequence

	Original report	Recent works that have benefited from the genome sequence
Ageing and cell degeneration	[40,103]	[104-106]
Cell death	[79]	[104,107]
Self/non-self recognition (vegetative incompatibility and hyphal interference)	[76,79]	[65]
Mating type and inter-nuclear recognition	[108]	[109]
Cell differentiation and cell signaling in filamentous fungi	[110]	[111]
Sexual reproduction in fungi	[21]	[64,111]
Mechanism of meiosis	[22,112]	
Meiotic drive	[113]	
Translation accuracy determinants and role	[114]	[115]; this paper
Mitochondrial physiology	[116,117]	[105]
Peroxisomal physiology and function	[118]	[119]
Prions and other protein-based inheritance	[120,121]	[106]
Biomass conversion	This paper	
Secondary metabolism		[122]

**Table 2:** Comparison between *P. anserina *and *N. crassa *biology

	*P. anserina *[80]	*N. crassa *[123]
**Ecology**		
Habitat	Restricted on dung of herbivores	Prefers plants killed by fire
	Always small biotopes and high competition	Often large biotopes and low competition
Distribution	Worldwide	Prefers hot climate
		
**Vegetative growth**		
Growth rate	Average (7 mm/d)	High (9 cm/d)
Ageing syndrome	Senescence in all investigated strains	Mostly immortal with some ageing strains
Hyphal interference	Present	Not yet described
Major pigments	Melanins (green)	Carotenoids (orange)
		
**Reproduction**		
Asexual reproduction	None	Efficient with germinating conidia
Sexual generation time	One week	Three weeks
Mating physiology	Pseudohomothallic	Strict heterothallic
Ascospore dormancy	No	Yes
Ascospore germination trigger	Passage through digestive track of herbivores in nature (on low nutrient media containing ammonium acetate in the laboratory)	60°c heat shock or chemicals (for example, furfural)
		
**Gene inactivation processes**		
RIP	Not efficient	Very efficient
MSUD	Not yet described	Efficient
Quelling	Not yet described	Efficient

Features and references pertaining to the biology of both fungi can be found at the corresponding reference.

## Results and discussion

### Acquisition, assembly and main features of the sequence

The genome of the laboratory reference S mat+ strain was sequenced using a whole-genome shotgun approach (see Materials and methods for a detailed explanation of the sequencing and assembly strategies). Ten-fold coverage permitted complete assembly of the mitochondrial genome as a single circular contig of about 95 kb and most of the nuclear genome (Table 3). The latter was assembled in 1,196 contigs clustered into 33 large scaffolds, comprising nearly all unique sequences, and 45 small scaffolds composed almost exclusively of transposon sequences, collectively totaling 35 Mb. Based on the frequency of sequence runs corresponding to the rDNA compared to that of unique sequences, we estimated that 75 rDNA units are present in the genome. With this assumption, the total sequence length of the genome is 35.5-36 Mb, a value somewhat superior to pulse field estimates [28,29]. Presently, all large scaffolds are assigned to a chromosome as defined by the genome map that now includes over 300 markers (see Materials and methods; Additional data file 1).

**Table 3:** Main features of the *P. anserina *genome

Genome features	Value
**Nuclear genome**	
Size	35.5-36 Mb
Chromosomes	7
GC percentage (total genome)	52.02
GC percentage in coding sequences	55.87
GC percentage in non-coding regions	48.82
tRNA genes	361
rDNA repeat number	75
Consensus rDNA repeat size	8192 pb
5S rRNAs	87
snRNA genes	14
snoRNA genes	13
Protein coding genes (CDSs)	10545
Percent coding	44.75
Average CDS size (min; max)	496.4 codons (10; 8,070)
Average intron number/CDS (max)	1.27 (14)
Average intron size (max)	79.32 nucleotides (2,503 nucleotides)
	
**Mitochondrial genome**	
Size	94,197 bp
Chromosome	1 (circular)
GC percentage	30%

**Figure 2:**
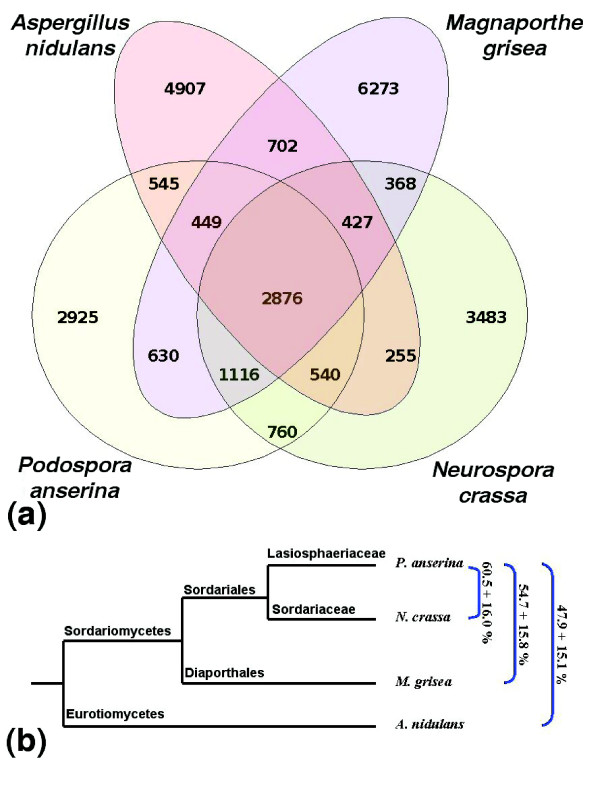
Orthologue conservation in some Pezizomycotina. **(a) **Venn diagram of orthologous gene conservation in four ascomycete fungi. The diagram was constructed with orthologous genes identified by the best reciprocal hit method with a cut-off e-value lower than 10^-3 ^and a BLAST alignment length greater than 60% of the query CDS. **(b) **Phylogenetic tree of the four fungal species. The average percentage of identity ± standard deviation between orthologous proteins of *P. anserina *and the three other fungi are indicated on the right.

The annotation strategy, described in the Materials and methods section, identified 10,545 putative coding sequences (CDSs), including two inteins [30]. 5S rRNA, tRNA, as well as several small nuclear RNAs (snRNAs) and small nucleolar RNAs (snoRNAs) were also identified. Statistics concerning the protein coding capacity of the *P. anserina *genome and the main features of the CDSs are indicated in Table 3. The present estimates of the coding capacity of *N. crassa *are 9,826 CDSs at the Broad Institute [31] and 9,356 CDSs at the Munich Information Center for Protein Sequences (MIPS) [32]. It remains to be established whether the higher coding capacity of *P. anserina *is real or due to the differences in strategies used to annotate the genomes of these fungi. We have searched for orthologous genes between *P. anserina*, *N. crassa*, *M. grisea *and *A. nidulans *by the best reciprocal hit method and found that these four fungi share a common core of 2,876 genes (Figure 2a). Comparison of the *P. anserina *CDSs with *N. crassa *orthologues (Figure 2b) indicates that they are, on average, 60.5 ± 16.0 percent identical, a value similar to the one calculated previously on a small sample [24]. The *P. anserina *CDSs were 54.7 ± 15.8% identical to *M. grisea *and 47.9 ± 15.1% to *A. nidulans *orthologues. The identities reflect the known phylogenetic relationship between these four pezizomycotina and are comparable to those found between species of saccharomycotina [9].

### The expressed sequence tag database analysis

In addition to genomic DNA sequencing, a collection of 51,759 cDNAs was sequenced. These originate from libraries constructed at different stages of the *P. anserina *life cycle (Table 4). The resulting expressed sequence tags (ESTs) were mapped on the genomic sequence to help with the annotation but also to gain insight into the transcriptional ability of *P. anserina*. As seen in Table 4, these cDNAs confirmed 5,848 genes. However, we detected alternative splicing events in 3.8% of the clusters. This suggests that the *P. anserina *proteome might be more complex than concluded from the present annotation. Of interest is the presence of 668 transcribed regions without obvious protein-coding capacity (designated here as 'non-coding transcripts'). Some of these produce ESTs that are spliced, poly-adenylated or present in multiple copies, suggesting that they originate from true transcription units. Although some genes may have been miss-called during annotation, these transcription units may correspond to transcriptional noise, code for catalytic/regulatory RNA or reflect polycistronic units coding for small peptides as described recently [33,34]. Finally, we detected 45 antisense transcripts corresponding to 36 different CDSs. These transcripts might potentially be involved in proper gene regulation, as described for the *S. cerevisiae PHO5 *gene [35]. In large scale analyses of *Fusarium verticilloides *[36] and *S. cerevisiae *[37] ESTs, similar arrays of alternatively spliced, 'non-coding' and antisense transcripts were detected, suggesting that the production of these 'unusual' transcripts is, in fact, a normal situation in ascomycete fungi, as described for other eukaryotes [38].

**Table 4:** EST analysis

				Alternatively spliced transcripts				
						
	Number of sequenced cDNA clones	Number of clusters	Confirmed genes*	Exon cassette	Alternative splice site	Retained intron	Non-coding transcripts not covering a predicted CDS	Antisense transcripts
**Bank**								
Mycelium grown for 48 h	27,291	6,054	5,780	1	155	137	322	19
Young perithecia of less than 48 h	7,695	2,392	2,236	2	46	55	258	12
Perithecia older than 48 h	7,814	2,373	2,088	2	26	51	440	4
Ascospores 20 h after germination trigger	5,570	1,589	1,502	0	29	28	125	3
Senescent mycelium	1,136	718	665	0	10	9	59	4
Incompatible mycelium	1,133	514	474	1	7	6	54	1
Rapamycin induced mycelium	1,120	593	543	1	3	11	68	2
								
**All databanks**	51,759	6,618	5,848	5	80	167	668	36

*Cluster covering a CDS.

### Genes putatively expressed through frame-shift or read-through

During the manual annotation of the genome, we detected 14 genes possibly requiring a frame-shift or a read-through to be properly expressed (Additional data file 2). In all cases, sequencing errors were discounted. In addition, ESTs covering putative read-through or frame-shift sites confirm six of them. Some of the putative frame-shifts and read-throughs detected could correspond to first mutations that will lead to pseudogene formation. However, four sites seem conserved during evolution, arguing for a physiological role. One of the putative -1 frame-shift sequences is located in the Yeti retrotransposon, a classic feature of this type of element. The 13 others affect genes coding for cellular proteins. Factors involved in the control of translation fidelity and affecting rates of frame-shift and read-through have been studied in *P. anserina *and shown to strongly impact physiology [39-42]. To date, the reasons for these effects are not known. None of the components responsible for insertion of selenocysteine are found in the *P. anserina *genome, excluding a role in the observed phenotypes of the non-conventional translation insertion of this amino acid, which takes place at specific UGA stop codons [43]. Similarly, no obvious suppressor tRNA was discovered in the genome.

### Synteny with *N. crassa*

**Figure 3:**
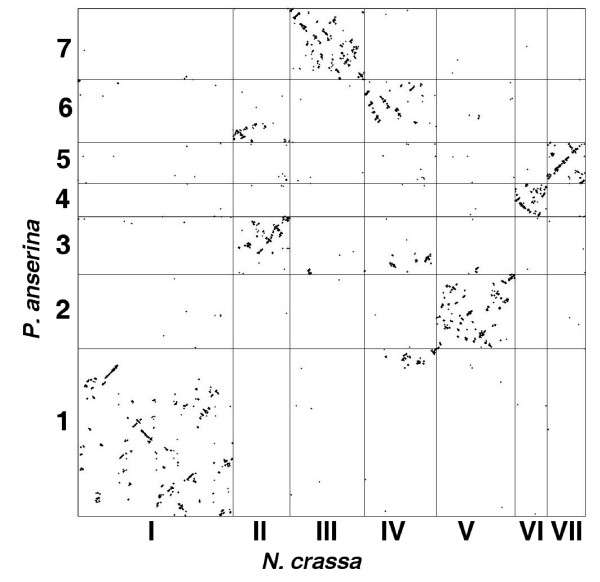
Genome-wide comparison of orthogolous genes of *N. crassa *(x-axis) and *P. anserina *(y-axis). Each dot corresponds to a couple of orthologous genes. The lines delimit the chromosomes. The scale is based on the number of orthologous genes per chromosome.

**Figure 4:**
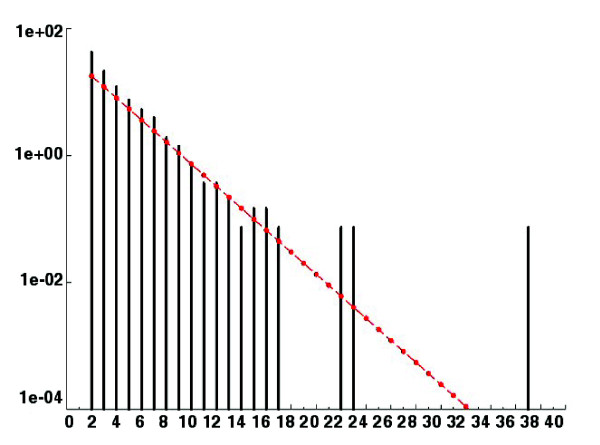
Size distribution of synteny block between *P. anserina *and *N. crassa*. Block size is given on the x-axis and frequency on the y-axis. Black bars indicate the actual value, and the red line shows the theoretical curve expected in the case of the random break model. The two distribution functions are not statistically different (Kolmogorov-Smirnov test, *p *>> 5%).

We have explored in more detail the synteny between orthologous genes in the *P. anserina *and *N. crassa *genomes (Figures 3 and 4). Synteny was defined as orthologous genes that have the same order and are on the same DNA strand. As observed for other fungal genomes [18,44], extensive rearrangements have occurred since the separation of the two fungi. However, most of them seem to happen within chromosomes since a good correlation exists between the gene contents of many chromosomes, even though a few translocations are detected (Figure 3). For example, most of *P. anserina *chromosome 1 corresponds to *N. crassa *chromosome I except for a small part, which is translocated to the *N. crassa *chromosome IV. Within the chromosomes, numerous rearrangements have occurred, compatible with the prevalence of small inversions in fungal genome evolution as observed previously between genes of saccharomycotina (hemiascomycetous) yeasts [45]. The size of the synteny blocks loosely follows an exponential decrease (Figure 4), compatible, therefore, with the random breakage model [46], suggesting that most breaks occur randomly, as observed for genome evolution in Aspergilli [18]. However, in both Aspergilli and saccharomycotina yeasts, blocks of synteny have been dispersed among the various chromosomes [18,47], unlike what is observed between *P. anserina *and *N. crassa*. This discrepancy of genome evolution between the three groups of fungi might stem from the fact that *P. anserina *and *N. crassa *have likely had a long history of heterothallism, whereas Aspergilli and saccharomycotina yeasts are either homothallic, undergo a parasexual cycle or switch mating types. In heterothallics, the presence of interchromosomic translocation results in chromosome breakage during meiosis and, hence, reduced fertility. On the contrary, homothallism, parasexualilty or mating-type switching may allow translocation to be present in both partners during sexual reproduction and, therefore, have fewer consequences on fertility. Additionally, meiotic silencing of unpaired DNA (MSUD), an epigenetic gene silencing mechanism operating in *N. crassa *[48], abolishes fertility in crosses involving rearranged chromosomes in one of the partners.

Interestingly, the largest synteny block between *P. anserina *and *N. crassa*, with 37 orthologous genes, encompasses the mating type, a region involved in sexual incompatibility. A similar trend in conserved synteny in the mating-type region has been observed in the genus *Aspergillus *[18]. This suggests that recombination may be inhibited in this region on an evolutionary scale. In both *P. anserina *and *N. crassa*, the mating-type regions are known to display peculiar properties. In *P. anserina*, meiotic recombination is severely repressed around the mating-type locus [49], as also described in *Neurospora tetrasperma *[50]. In *N. crassa*, MSUD is inhibited in the *mat *region [48]. However, recombination is not completely abolished around this locus. Indeed, between pairs of orthologous genes, a few species-specific CDSs were detected. These genes may come from *de novo *insertion or, alternatively, these species-specific genes have been lost in the other species. This lends credit to the hypothesis put forward to explain the mating-type region of *Cryptococcus neoformans *[51], in which the genetic incompatibility is driven by two genetically different sequences of 100 kb. In these regions, not only the mating-type regulatory genes are different, but also housekeeping genes. Inhibition of recombination at this locus may have driven the differential acquisition of genes by the two haplotypes within the same species. Note that on a longer evolutionary scale, inhibition of recombination cannot be detected because the synteny of the mating-type region of *P. anserina *with that of *M. grisea *or *A. nidulans *is absent or limited to very few genes.

### Repeated sequences in the *P. anserina *genome

**Figure 5:**
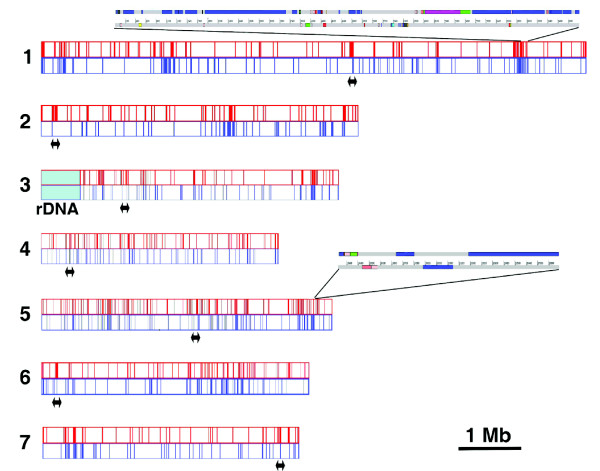
Repartition of transposons (top in red) and segmental duplications (bottom in blue) in the *P. anserina *genome. Chromosome numbering and orientation is that of the genetic map [85]. The double arrows indicate the putative centromere positions. Two regions have been expanded to show the interspacing of segmental duplications (in blue) with transposons (other colors); numbering refers to the nucleotide position with respect to the beginning of the scaffolds.

The pilot project that sequenced about 500 kb around the centromere of chromosome 5 revealed an apparent paucity in repeated sequences in *P. anserina *[24]. The draft sequence reported here confirms a paucity of repeats but not as much as suggested by the pilot project. In fact, repeats cover about 5% of the *P. anserina *genome (omitting the rDNA cluster). They can be divided into four categories: RNA genes (Table 3; see Materials and methods), true transposons (Additional data file 3), repetitive elements of unknown origin (Additional data file 3) and segmental duplications (Additional data file 4). Collectively, the transposons occupy about 3.5% of the genome. However, as many transposons border the sequence gaps present in the draft assembly, the actual percentage in the complete genome may be higher. This is about three times less than in the genomes of *M. grisea *[11] and *N. crassa *[16], close relatives of *P. anserina*. Most segmental amplifications are small (Additional data file 4), although one is 20 kb large. They occupy about 1.5% of the genome. An interesting feature of all these repeated sequences (except for the 5S RNA and tRNA genes) is that they are nested together (Figure 5), as previously described for *Fusarium oxysporum *transposons [52]. In particular, large parts of many chromosomes are almost devoid of these repeated sequences whereas chromosome 5 is enriched in repeats. Ironically, the pilot project sequenced a region of this chromosome 5 almost devoid of repeated sequences.

Nearly all copies of these repeated elements differ by polymorphisms, many of which appear to be caused by repeat induced point mutation (RIP). RIP is a transcriptional gene silencing and mutagenic process that occurs during the sexual dikaryotic stage of many pezizomycotina [53]. *P. anserina *displays a very weak RIP process [54,55]. It results, as in *N. crassa*, in the accumulation of C●G to T●A transitions in duplicated sequences present in one nucleus, and, therefore, 'ripped' sequences present a higher than average T/A content. However, although the RIP process acts in the *P. anserina *genome, it does not account for all the mutations found in these inactivated paralogues. For example, the copies of 'rainette', the last transposon to have invaded the *P. anserina *genome (Additional data file 3), differ by 30 polymorphic sites. Twenty-five of them (83%) were C●G versus T●A polymorphisms and may, therefore, be accounted for by RIP, while the five others (17%) cannot. A reciprocal ratio was observed in other instances as seen for the largest segmental triplication with two copies present on chromosome 5 and one on chromosome 1. The three members share a common region of about 9 kb. In this region they differ by numerous indels and in about 20% of their nucleotides. More precisely, in the 4,000 nucleotide-long core region where the three sequences can unambiguously be aligned, there are 1,341 polymorphic sites in which at least one sequence differs from the others. For 418 of them (31%), two members have a C●G polymorphism whereas the other has a T●A polymorphism, strongly suggesting that these polymorphisms may originate from RIP, whereas for the remaining 923 (69%), the variations are small indels or single nucleotide variations not accounted for by RIP. Therefore, in the case of rainette, RIP polymorphisms are foremost, whereas for the triplication, non-RIP polymorphisms are more frequent. This is compatible with a model in which RIP occurs first and is then followed by accumulation of other types of mutations.

Overall, these data suggest that *P. anserina *has experienced a fairly complex history of transposition and duplications, although it has not accumulated as many repeats as *N. crassa*. *P. anserina *possesses all the orthologues of *N. crassa *factors necessary for gene silencing (Additional data file 5), including RIP, meiotic MSUD [48] and also vegetative quelling, a post transcriptional gene silencing mechanism akin to RNA interference [56]. However, to date, no MSUD or quelling has been described in *P. anserina*, despite the construction of numerous transgenic strains since transformation was first performed [57]. Surprisingly, the DIM-2 DNA methyltransferase [58], the RID DNA methyltransferase-related protein [59] and the HP1 homolog necessary for DNA methylation [60] described in *N. crassa *are present in the genome of *P. anserina*. Although the *P. anserina *orthologues of these two proteins seem functional based on the analysis of the conserved catalytic motifs, no cytosine methylation has been reported to occur in this fungus [54]. A possibility would be that methylation is restricted to a specific developmental stage or genomic region that has not yet been investigated. Overall, the apparent absence (quelling and MSUD) or lack of efficiency (RIP) of these genome protection mechanisms in *P. anserina *questions their true impact on genome evolution, especially since this fungus contains less repeated sequences than *N. crassa*. Maybe the life strategy of *P. anserina *makes it less exposed to incoming selfish DNA elements, therefore diminishing the requirement of highly efficient gene silencing mechanisms. Supporting this assumption is the fact that, although heterothallic, formation of ascospores makes *P. anserina *pseudo-homothallic (Figure 1), with seemingly very little out-crossing [61], whereas *N. crassa *is strictly heterothallic and presents a low fertility in crosses between closely related strains [62].

### Gene evolution by duplication and loss in fungi

The detection of segmental duplications raised the question of whether new genes evolved through duplication in the lineage that gave rise to *P. anserina*. It is known that creating new genes through duplication in *N. crassa*, in which RIP is very efficient, is almost impossible [16]. On the contrary, RIP is much less efficient in *P. anserina*; in particular, RIP is absent in progeny produced early during the maturation of the fructifications [55]. In addition, the mutagenic effect of RIP is very slight since it has been estimated that at most 2% of cytosines are mutated when RIP affects duplicated sequences present on two different chromosomes [63]. We previously reported that some thioredoxin isoforms were encoded by a triplicated gene set in *P. anserina *as compared to *N. crassa *[64], showing that gene duplications can indeed generate new genes in *P. anserina*. However, thioredoxins are small proteins encoded by small genes. To test if large genes were duplicated, we performed a three-way comparison between the *P. anserina*, *N. crassa *and *M. grisea *putative CDSs and screened for *P. anserina *CDSs that show a best hit with another *P. anserina *CDS to the exclusion of proteins from *N. crassa *and *M. grisea*. Such CDSs may originate from duplication that occurred in the *P. anserina *lineage after its divergence from *N. crassa*. In this analysis, small genes were excluded because the putative candidates were selected on the basis of an e-value of less than 10^-190 ^in Blast comparison against the database containing the three predicted proteomes (as a consequence, the thioredoxin genes were not included in the set).

**Figure 6:**
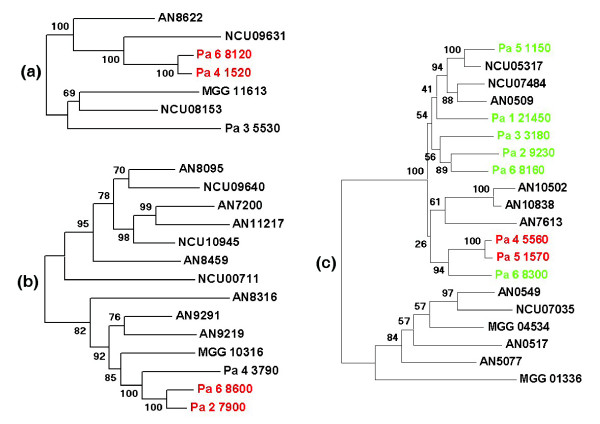
Gene gain and loss in fungal genomes. **(a-c) **Unrooted phylogenetic trees of putative alkaline phosphatase D precusors (a), putative HC-toxin efflux carrier proteins related to ToXA from *Cochliobolus carbonum *(b), and putative chitinases related to the killer toxin of *Kluyveromyces lactis *(c). The putative CDSs were aligned with ProbCons 1.10 [101] and manually edited to eliminate poorly conserved regions, resulting in alignment over 565, 544, 505 amino acids, respectively. Phylogenetic trees were constructed with Phyml 2.4.4 [102] under the WAG model of amino acid substitution. The proportion of variable sites and the gamma distribution parameters of four categories of substitution rate were estimated by phyml. For each tree, we performed 100 boostrap replicates. The recently duplicated *P. anserina *paralogues are highlighted in red and the divergent duplication of chitinases in green. Trees with similar topologies and statistical support (1,000 boostrap replicates) were recovered with the neighbor joining method. Especially, recent duplication of Pa_4_1520/Pa_6_8120, Pa_2_7900/Pa_6_8600 and Pa_4_5560/Pa_5_1570 as well as the distinction of the two subfamilies of chitinases were recovered with 100% confidence. AN, *A. nidulans*; MGG, *M. grisea*; NC, *N. crassa*; Pa, *P. anserina*.

To confirm that the candidates recovered indeed originated from recent duplications, phylogenetic trees were constructed with the CDSs from *P. anserina*, *N. crassa*, *M. grisea *and additional fungal CDSs. In some instances, the trees confirmed a recent duplication event in the *P. anserina *lineage after the split between *P. anserina *and *N. crassa*, because the phylogenetic analysis clustered the *P. anserina *paralogues with high statistical confidence. Figure 6 shows the trees obtained for three such couples of paralogues, for example, genes coding for putative alkaline phosphatase D precursors (Pa_4_1520 and Pa_6_8120; Figure 6a), putative HC-toxin efflux carrier proteins related to ToXA from *Cochliobolus carbonum *(Pa_2_7900 and Pa_6_8600; Figure 6b) and putative chitinases related to the killer toxin of *Kluyveromyces lactis *(Pa_4_5560 and Pa_5_1570; Figure 6c). Overall, our analysis detected an initial set of 33 putative duplicated gene families, including the het-D/E gene family, whose evolutionary history has been reported elsewhere [65]. Among these, at least nine (including the het-D/E genes) have duplicated recently. However, some additional recent duplication events may have occurred but are not supported with sufficient statistical confidence to differentiate between recent duplications followed by rapid divergence, and ancient duplications (see Figure 6c for an example of such duplications with putative chitinases). The fact that large genes may duplicate in *P. anserina *is not contradictory to the presence of RIP, since if RIP may inactivate genes when efficient, it can accelerate gene divergence when moderately efficient, as described for the het-D/E family [65].

The phylogenetic analyses of the multigene families suggest that gene loss may also have occurred during fungal evolution. The putative chitinases related to the killer toxin of *K. lactis *provide a clear example of this situation. *N. crassa *and *M. grisea *have two paralogues, whereas *P. anserina *has eight. The phylogenetic tree including the ten paralogues present in *A. nidulans *(Figure 6c) suggests that these proteins can be grouped into two families. Surprisingly, the *P. anserina *proteins cluster in one subfamily, whereas the *M. grisea *proteins cluster in the other, indicating differential gene losses. In *P. anserina*, even if Pa_4_5560 and Pa_5_1570 seem to have duplicated recently, this is not as clear for the other members since they are not very similar. They may result from ancient gene duplications or from recent duplications followed by rapid evolution, possibly thanks to RIP. Evolution of this family seems thus to proceed by a complex set of gain and loss at various times. The same holds true for polyketide synthase (PKS) genes. Seven PKSs were reported for *N. crassa *[16], while *M. grisea *has 23 [11], and we identified 20 PKS genes for *P. anserina*. A comparison of all these PKSs (data not shown) indicates a complex evolution process in which *N. crassa *has probably lost most of its PKSs and the two other fungi present several duplications yielding very different copies. Again, this does not permit us to establish whether the duplications are ancient or recent but followed by intense divergence. See also below for additional examples of losses and amplifications of genes involved in carbon source degradation.

Such gene losses may be frequent events in filamentous ascomycete. As seen in Figure 2a, *P. anserina*, *M. grisea *and *A. nidulans *share 1,624 genes that seem to be lacking in *N. crassa *(among these, 449 are present in the three fungi, 630 in both *P. anserina *and *M. grisea*, and 545 in both *P. anserina *and *A. nidulans*), even though *M. grisea *and *A. nidulans *are more distantly related to *P. anserina *than is *N. crassa *(Figure 2b). Although some genes may have evolved beyond recognition specifically in *N. crassa*, the most parsimonious explanation is that *P. anserina *has retained many genes that *N. crassa *has lost. Similarly, *N. crassa*, *M. grisea *and *A. nidulans *share 1,050 genes that are absent in *P. anserina*. Therefore, we tentatively suggest that genomes from sordariomycetes may be shaped by more gene loss and gene duplications than anticipated by the presence of RIP. Similar rates of gene loss in filamentous ascomycetes have recently been demonstrated [66].

### Carbon catabolism

In nature, *P. anserina *lives exclusively on dung of herbivores. In this biotope, a precise succession of fungi fructifies [67]. An explanation put forward to account for this succession is nutritional. The first fungi to appear feed preferably on simple sugars, which are easy to use, followed by species able to digest more complex polymers that are not easily degraded. Indeed, the mucormycotina zygomycetes, which are usually the first ones to fructify on dung, prefer glucose and other simple sugars as carbon sources. They are followed by ascomycetes that use more complex carbohydrates such as (hemi)cellulose but rarely degrade lignin. The succession ends with basidiomycetes, some of which can degrade lignin to reach the cellulose fiber not available to other fungi [68-70].

Usually, *P. anserina *fructifies in the late stage of dung decomposition [67]. This late appearance of the *P. anserina *fruiting body is hard to correlate with slow growth of the mycelium and delay in fructification since in laboratory conditions ascospore germination occurs overnight and fruit body formation takes less than a week. However, *P. anserina *harbors unexpected enzymatic equipment, suggesting that it may be capable of at least partly degrading lignin, which concurs with the nutritional hypothesis (Table 5). It includes a large array of glucose/methanol/choline oxidoreductases [71], many of which are predicted to be secreted, two cellobiose dehydrogenases, a pyranose oxidase, a galactose oxidase, a copper radical oxidase, a quinone reductase, several laccases and one putative Lip/Mn/Versatile peroxidase. Enzymes homologous to these CDSs are known to produce or use reactive oxygen species during lignin degradation [68-70]. This ascomycete fungus may thus be able to access carbon sources normally available mainly to basidiomycetes. Interestingly, *P. anserina *is closely related to xylariales, a group of ascomycete fungi that seems to contain true white rot fungi capable of degrading lignin [72]; also, *P. anserina *has the most complete enzymatic toolkit involved in lignin degradation when compared to the three other ascomycetes included in Table 5. The comparison with *N. crassa *is particularly striking. This is in line with the fact that *N. crassa *in its less competitive biotope may have access to more easily digestible carbon sources.

**Table 5:** CDSs putatively involved in lignin degradation

		*P. anserina*				
					
	Reference		Secretion*	*N. crassa*	*M. grisea*	*A. nidulans*
GMC oxidoreductases	[124]	Pa_0_190	+	NCU09798.3	MGG_07580.5	AN2175.3
		Pa_5_1280	+?	NCU04938.3	MGG_07941.5	AN7998.3
		Pa_1_15920	+	NCU01853.3	MGG_08438.5	AN4006.3
		Pa_5_4870	-	NCU07113.3	MGG_10479.5	AN3229.3
		Pa_4_5130	+	NCU09024.3	MGG_05055.5	AN4212.3
		Pa_5_5180	+?	NCU08977.3	MGG_10933.5	AN9011.3
		Pa_6_6430	-?		MGG_11204.5	AN7267.3
		Pa_1_23060	+		MGG_12623.5	AN9348.3
		Pa_6_7550	+		MGG_12626.5	AN6445.3
		Pa_2_7270	+		MGG_14477.5	AN7812.3
		Pa_1_470	+		MGG_02127.5	AN1093.3
		Pa_5_9820	-		MGG_09072.5	AN2704.3
		Pa_6_1080	+		MGG_06596.5	AN3531.3
		Pa_1_24480	-		MGG_00779.5	AN7408.3
		Pa_0_340	-		MGG_02371.5	AN1429.3
		Pa_4_880	+		MGG_10948.5	AN8329.3
		Pa_7_4250	+		MGG_11676.5	AN8547.3
		Pa_5_12190	+		MGG_13253.5	AN3206.3
		Pa_4_4320	+?		MGG_11317.5	AN0567.3
		Pa_3_11130	+?		MGG_13583.5	AN3960.3
		Pa_1_21970	-		MGG_08487.5	AN7890.3
		Pa_3_1060	+		MGG_09189.5	AN7832.3
		Pa_7_4780	+		MGG_07569.5	AN7056.3
		Pa_0_440	+			
		Pa_5_4150	+			
		Pa_6_11490	+			
		Pa_5_12200	+			
		Pa_5_13040	-?			
		Pa_6_11360	-			
Cellobiose dehydrogenases	[125]	Pa_7_2650	+	NCU00206.3	MGG_11036.5	AN7230.3
		Pa_0_280	+	NCU05923.3	MGG_13809.5	
Pyranose oxidases	[126]	Pa_6_8060	?	-	-	AN5281.3
Galactose oxidases	[127]	Pa_1_18310	+	NCU09209.3	MGG_10878.5	-
					MGG_12681.5	
Copper radical oxidases	[128]	Pa_1_7300	+	NCU09267.3	MGG_01655.5	-
					MGG_05865.5	
Quinone reductase	[129]	Pa_1_6390	-?	NCU02948.3	MGG_01569.5	AN0297.3
Laccases	[130]	Pa_5_1200	+?	NCU04528.3	MGG_09102.5	AN0901.3
		Pa_5_4660	+	NCU05113.3	MGG_08523.5	AN6635.3
		Pa_7_4200	+	NCU05604.3	MGG_07771.5	AN0878.3
		Pa_5_9860	+	NCU09279.3	MGG_02876.5	AN6830.3
		Pa_7_3560	+?	NCU02201.3	MGG_09139.5	AN5397.3
		Pa_6_10630	+	NCU00526.3	MGG_05790.5	AN9170.3
		Pa_1_15470	+	NCU07920.3	MGG_11608.5	
		Pa_6_7880	-	NCU09023.3	MGG_08127.5	
		Pa_1_16470	+		MGG_13464.5	
		Pa_5_4140	?			
lip/Mn/versatile peroxidases	[70,131]	Pa_1_5970	?	-	MG_07790.5	-
					MGG_03873.5	

*Orthologues were identified by the best reciprocal hit method. Putative secretion was evaluated by searching for the presence of a secretion signal peptide with Interproscan or by evaluating the most probable localization with WolfPSORT. In most instances, both methods yielded the same result. '+', protein likely secreted; '-', protein likely not secreted; '?', no firm conclusion could be reached as to the actual localisation. GMC, glucose/methanol/choline.

As mentioned above, *P. anserina *is considered a late growing ascomycete on herbivorous dung. This suggests that the fungus is likely to target lignocellulose as a carbon source, since most hemicellulose and pectin would probably be consumed by zygomycetes and early ascomycetes. A close examination of the genome sequence of *P. anserina *for the presence of carbohydrate active functions (Additional data file 6) and a comparison with the genome sequence of other fungi confirmed the adaptation capacity of *P. anserina *to growth on lignocellulose. The total number of putative glycoside hydrolases (GHs), glycoside transferases, polysaccharide lyases (PLs) and carbohydrate esterases (CEs) are similar to those of other ascomycetes, such as *A. niger *[20] and *M. grisea *[73], but *P. anserina *has the highest number of carbohydrate-binding modules (CBMs) of all the fungal genomes sequenced to date. Despite possessing similar numbers of putative enzymes, the distribution of the possible enzyme functions related to plant cell wall degradation (Table 6) is significantly different in *P. anserina *from that of other fungi. *P. anserina *has the largest fungal set of candidate enzymes for cellulose degradation described to date. This is particularly remarkable in GH family 61 (GH61) with 33 members, two-fold higher than the phytopathogen ascomycete *M. grisea *and the white rot basidiomycete *P. chrysosporium*. Similar patterns are visible for other cellulose-degrading families (for example, GH6, GH7, GH45) and in the high number of CBM1 (possibly cellulose-binding) modules found, which are only equivalent to the sets of *P. chrysosporium *and *M. grisea*.

**Table 6:** Comparison of relevant CAZy family content related to plant cell wall polysaccharide degradation

CAZy family	Main substrate	*P. anserina*	*N. crassa*	*M. grisea*	*A. nidulans*	*A. niger*	*P. chrysosporium*
**Plant cell wall degradation**							
GH1	Cellulose/hemicellulose	1	1	2	3	3	2
GH2	Hemicellulose	8	5	6	10	6	2
GH3	Cellulose/hemicellulose/xylan	11	9	19	21	17	11
GH5	Cellulose	15	7	13	16	10	20
GH6	Cellulose	4	3	3	2	2	1
GH7	Cellulose	6	5	6	3	2	9
GH10	Xylan	9	4	5	3	1	6
GH11	Xylan	6	2	5	2	4	1
GH12	Cellulose/xylan	2	1	3	1	3	2
GH28	Pectin	0	2	3	10	21	4
GH29	Hemicellulose	0	0	4	0	1	0
GH35	Hemicellulose	1	2	0	4	5	3
GH36	Hemicellulose	1	1	2	4	3	0
GH43	Hemicellulose	13	7	19	18	10	4
GH45	Cellulose	2	1	1	1	0	0
GH51	Hemicellulose	1	1	3	3	3	2
GH53	Hemicellulose	1	1	1	1	2	1
GH54	Hemicellulose	0	1	1	1	1	0
GH61	Cellulose	33	14	17	9	7	15
GH62	Hemicellulose	2	0	3	2	1	0
GH67	Xylan	1	1	1	1	1	0
GH74	Hemicellulose	1	1	1	2	1	4
GH78	Pectin	1	0	1	9	8	1
GH88	Pectin	0	0	1	3	1	1
GH93	Hemicellulose	3	2	1	2	0	0
GH94	Cellulose	1	1	1	0	0	0
GH95	Hemicellulose	0	0	1	3	2	1
GH105	Pectin	0	1	3	4	2	0
							
PL1	Pectin	4	1	2	9	6	0
PL3	Pectin	2	1	1	5	0	0
PL4	Pectin	1	1	1	4	2	0
PL9	Pectin	0	0	0	1	0	0
PL11	Pectin	0	0	0	1	0	0
							
CE1	Xylan	14	7	10	4	3	5
CE8	Xylan	1	1	1	3	3	2
CE12	Xylan	1	1	2	2	2	0
							
CBM1	Cellulose	28	20	22	7	8	30
							
**Other relevant families**							
GH18	Chitin	20	12	14	20	14	11
GH32	Sucrose/inulin	0	1	5	2	6	0
CBM18	Chitin	30	3	29	19	13	1

Strikingly, *P. anserina *also has an increased potential for xylan degradation, with abundant enzyme sets in families GH10 and GH11, together with a relative abundance of exo-acting enzymes in families GH3 and GH43. Interestingly, no α-fucosidases of families GH29 and GH95 are found, suggesting a depletion of xyloglucan prior to growth of *P. anserina*. During the stage at which *P. anserina *grows in dung, significant amounts of cellulose, but also xylan, are still available. Xylan can be cross-linked to lignin through ferulic acid [74] or 4-O-methyl-glucuronic acid [75]. In light of the potential of *P. anserina *for lignin degradation, it is conceivable that this fungus particularly consumes lignin-linked xylan that could not be degraded by 'earlier' growing organisms that lack a lignin-degradation system. The relatively high number of putative CE1 acetyl xylan and feruloyl esterases found in *P. anserina *by comparison with other fungi correlates with this hypothesis.

In contrast to the increased potential for cellulose and xylan degradation, a significantly weak potential for pectin degradation was observed for *P. anserina*. No members of GH28 (containing pectin hydrolases) were detected in the genome and only a single α-rhamnosidase (GH78). In comparison, *A. niger *contains 21 GH28 members and 8 GH78 members [20]. The number of putative pectin lyases is also much smaller than that observed for *A. niger*. The auxiliary activities of GH88 and GH105, likely to act on pectin lyase degradation products, are equally absent from *P. anserina *while present in all pectin-degrading organisms (Table 6). The absence of the potential to degrade sucrose and inulin is concluded from the lack of enzymes in the GH32 family. This also correlates with the low capacity of *P. anserina *to grow on rapidly degradable carbohydrates that are most likely depleted by 'earlier' organisms. Furthermore, the large number of GH18 and CBM18 modules, 20 and 30 respectively, could indicate that *P. anserina *has the ability to degrade exogenous chitin and possibly to depend on available fungal cell material (derived from the set of fungi that grow earlier on dung of herbivores and that *P. anserina *may kill by hyphal interference [76]).

**Figure 7:**
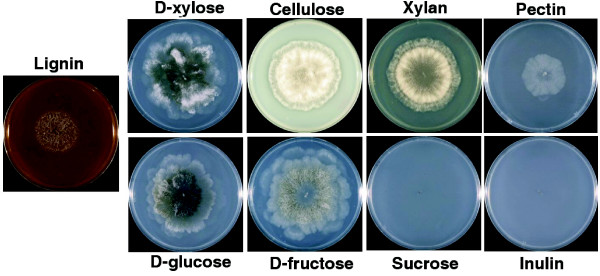
Carbohydrate utilization in *P. anserina*. Cultures were incubated for one week with 1% of the indicated compounds as carbon source.

To evaluate whether the enzymatic potential reflects the ability of *P. anserina *to degrade plant polymeric substrates, growth was monitored on minimal medium plates containing lignin, cellulose, beech wood xylan, apple pectin, inulin and 25 mM sucrose, D-glucose, D-fructose or D-xylose (Figure 7). *P. anserina *did grow on lignin, indicating that it is able to degrade lignin. However, it is suspected that in nature lignin degradation, an energy consuming process, may not be to obtain a carbon source, but mainly to gain access to the (hemi-)cellulose. Growth on cellulose, xylan and D-xylose was significantly faster than on pectin, which agrees with the enzymatic potential based on the genome sequence as described above. No growth was observed on inulin or sucrose, while efficient growth was observed on D-fructose and D-glucose. This is in agreement with the absence of genes required to degrade sucrose and inulin from the genome of *P. anserina*. Overall, these data suggest that *P. anserina *has all the enzymatic complement necessary to efficiently scavenge the carbohydrates it encounters in its natural biotope. Selection has in fact evolved its genome to deal efficiently with these carbon sources, first by duplicating genes involved in cellulose degradation, as shown by the high number of GH61 CDSs, and second by deleting genes required to use carbon sources not commonly encountered (for example, pectin, inulin, and sucrose). This demonstrates the high environmental pressure on evolution as well as the high level of specialization that occurs in the fungal kingdom.

## Conclusion

Our analysis of the genome sequence of *P. anserina*, a saprophytic model ascomycete, provides new insights into the genomic evolution of fungi. EST analysis indicates that similar to other eukaryotes, the transcription machinery generates a large array of RNAs with potential regulatory roles. Functional characterization of these RNAs might be one of the most interesting perspectives of this study. Strikingly, in addition to abundant inversions of chromosome segments and gene losses, substantial gene duplications were uncovered. Since this fungus displays a mild RIP, these findings allow us to ask whether the RIP process, when relatively inefficient, might be more of a genome evolution tool rather than a genome defense mechanism.

Moreover, availability of the genome sequence has also already permitted the development of new tools that will bolster research in *P. anserina*. The polymorphic markers designed to plot scaffolds onto the genetic map are now successfully used for positional cloning. Gene deletion is facilitated thanks to the availability of the *PaKu70 *mutant strain, which greatly enhanced homologous recombination [77], similarly to the deletion of the homologous gene in *N. crassa*, *mus-51 *[78]. The identification of the *PaPKS1 *gene by a candidate gene approach permits us to envision the design of new genetic tools based on mycelium or ascospore color [63]. The design of microarrays for transcriptome analyses is under way.

As for other saprophytic fungi, the *P. anserina *genome sequence has opened new avenues in the comprehensive study of a variety of biological processes. Of importance is the novel discovery of a large array of *P. anserina *genes potentially involved in lignin and cellulose degradation, some of which may be used for biotechnology applications. It also demonstrates how *P. anserina *is well adapted at the genome level to its natural environment, which was confirmed by the analysis of growth profiles. This result emphasizes the necessity to study several less well-tracked organisms in addition to those well known in the scientific community, as these may yield unexpected new insights into biological phenomena of general interest.

## Materials and methods

### Strains and culture conditions

The sequenced strain is the S mat+ homokaryotic strain [79]. Culture conditions for this organism were described [61], and currently used methods and culture media can be accessed at the *Podospora anserina *Genome Project web site [80].

### Genomic DNA library construction

Nuclear genomic DNA was extracted and separated from mitochondrial DNA as described [81]. Residual mitochondrial DNA present in the preparation was sufficient to allow sequencing of the full mitochondrial DNA circular chromosome. Construction of plasmid DNA libraries was made at Genoscope. The construction of the bacterial artificial chromosome (BAC) library is described in [24].

### Construction of cDNA library

Two strategies were used to construct the cDNA libraries. First, a mycelium library was constructed in the yeast expression vector pFL61 [82]. Total RNA was extracted from the s wild-type strain (mat-) and polyA^+ ^RNA was purified twice on oligo (dT)-cellulose columns (mRNA purification kit, Amersham Pharmacia Biotech, GE Healthcare Bio-Sciences AB, Uppsala, Sweden). Anchored dT25 primers were used to obtain double-stranded DNA (cDNA kit, Amersham Pharmacia Biotech, GE Healthcare Bio-Sciences AB, Uppsala, Sweden). Three cDNA libraries, corresponding to three ranges of molecular weight cDNA (0.2-1 kb, 1-2.5 kb, > 2.5 kb) were cloned using BstX1 adaptators in the pFL61 vector between the 5' (promoter) and 3' (terminator) sequences of the *S. cerevisiae pgk1 *gene as described previously [82].

Second, total RNA obtained under various physiological conditions (Table 4) was extracted as described [83], using the 'RNeasy Maxi Kit' (Qiagen, Germantown, MD, USA). PolyA^+ ^mRNAs were extracted with the 'Oligotex mRNA Maxi Kit' (Qiagen), reverse transcribed and cloned with the 'cloneMiner cDNA library construction Kit' into plasmid pDONR222 (Invitrogen, Carlsbad, CA, USA).

### Sequencing and assembly strategy

The genome of *P. anserina *was sequenced using a 'whole genome shotgun and assembly' strategy. We generated 510,886 individual sequences from two plasmid libraries of 3.3 and 12 kb insert sizes, and from one BAC library of about 90 kb insert size. This corresponds to genome coverage of 9.7-fold. The reads were automatically assembled using Arachne [84], and the initial assembly was improved by eliminating small redundant scaffolds. Additionally, in cases when the genetic map indicated the proximity of two scaffolds (see below), we joined them if there was some additional read pair information between them that was not used by Arachne. Some inter-contig gaps were also filled by placing a contig between two other contigs when matches and read pair information existed and were coherent. The final automatic assembly consisted of 2,784 contigs of N50 size 43 kb, grouped in 728 scaffolds of N50 size 638 kb, for a total genome size (without gaps) of 35.7 Mb. Manual sequence gap filling and removal of contigs corresponding to rDNA genes permitted the decrease of scaffolds and contig numbers to 1,196 contigs clustered into 78 scaffolds.

To connect the genome sequence with the genetic map [85], two approaches were followed. First, sequenced genes, whose positions on the genetic map were known, were mapped by searching the corresponding sequence in the scaffolds, enabling the attribution of some scaffolds to known chromosomes. Second, potential molecular polymorphic markers (microsatellites, minisatellites and indels) were searched and their polymorphisms were assessed in geographic isolates D, E M, T and U. It rapidly appeared that strain T was the genetically most distant strain from strain S, since about three-quarters of tested markers were actually polymorphic between the two strains. A cross between the T and S strains was set up and 51 homokaryotic progenies from this cross were assayed for 120 polymorphic sites scattered onto the 36 largest scaffolds that represented all the coding parts of the genome (except for one putative CDS). Linkage analysis made it possible to define seven linkage groups that were matched with the chromosomes thanks to the already known genes mapped on the sequence by the first approach. Additional polymorphic markers were then used to confirm local assembly, resulting in the new genome map, which contain 325 markers (Additional data file 1). No discrepancy was observed between the established genetic map, the newly defined linkage groups and the sequence assembly. Presently, all but one CDS-containing scaffold are attributed to a chromosome position, although in a few cases orientation of some scaffolds within the chromosome could not be accurately defined because of their small size. One 33 kb scaffold containing one predicted CDS as well as small scaffolds exclusively made up of repeated sequences are presently not mapped. Collectively, they represent about 1% of the genome.

### EMBL accession numbers

Chromosome 1: CU633438; CU633901; CU633867; CU633899; CU633445; CU633897. Chromosome 2: CU633446; CU640366; CU633447. Chromosome 3: CU633448; CU633447; CU633453. Chromosome 4: CU633454; CU633455; CU633456; CU633895. Chromosome 5: CU633457; CU633458; CU633459; CU633866; CU633871; CU607053; CU633461, CU633870, CU633865, CU633876. Chromosome 6: CU633898; CU638744; CU633463, CU633872. Chromosome 7: CU633900; CU633464; CU633873.

### Annotation and analysis of genomic sequences

CDSs were annotated by a combination of semi-automatic procedures. First, *P. anserina *open reading frames longer than 20 codons that are evolutionary conserved in *N. crassa *were retrieved by TBLASTN analysis. Candidates with an e-value lower than 10^-18 ^were conserved as hypothetical exons. Exons separated by less than 200 nucleotides were merged into putative CDSs and putative introns were predicted thanks to the *P. anserina *consensus sequences defined in the pilot project [24]. Then, 5' and 3' smaller exons were searched by the same procedure except that open reading frames longer than five codons surrounding putative CDSs were analyzed by BLAST with the homologous *N. crassa *region. Candidates with an e-value lower than 10^-5 ^were conserved and added to the putative CDSs. CDS and intron predictions were edited with Artemis [86] and manually corrected after comparison with available ESTs. Finally, *ab initio *prediction with GeneID [87] using the *N. crassa *and *Chaetomium globosum *parameter files were performed on regions devoid of annotated features. Manual verification was then applied to improve prediction. This resulted in the definition of 10,545 putative CDSs.

A canonic rDNA unit was assembled. A junction sequence between the left arm of chromosome 3 and an rDNA unit was observed, confirming the position of the cluster on this chromosome based on pulse field electrophoresis data [28]. On the other end of the cluster a junction between an incomplete rDNA repeat and CCCTAA telomeric repeats [88] was detected showing that the cluster is in a subtelomeric position. Similar to the previously investigated filamentous fungi [89], 5S rRNAs were detected by comparison with the *N. crassa *5S genes. They are encoded by a set of 87 genes, including 72 full-length copies dispersed in the genome. tRNAs were identified with tRNAscan [90]. A total of 361 genes encode the cytosolic tRNA set, which is composed of 48 different acceptor families containing up to 22 members. This set enabled us to decode the 61 sense codons with the classical wobble rule. Other non-coding RNAs were detected with a combination of the Erpin [91], Blast [92] and Yass [93] programs. Homology search included all RNAs contained in the RFAM V.8 [94] and ncRNAdb [95] databases. Any hit from either program with an e-value below 10^-4 ^was retained, producing a list of 28 annotated non-coding RNA genes or elements, including 12 spliceosomal RNAs, 15 snoRNAs (mostly of the C/D box class) and one thiamine pyrophosphateriboswitch.

### Alignment of EST sequences on the *P. anserina *genome

A two-step strategy was used to align the EST sequences on the *P. anserina *genome. As a first step, BLAST [92] served to generate the alignments between the microsatellite repeat-masked EST sequences and the genomic sequence using the following settings: W = 20, X = 8, match score = 5, mismatch score = -4. The sum of scores of the high-scoring pairs was then calculated for each possible location, then the location with the highest score was retained if the sum of scores was more than 1,000. Once the location of the transcript sequence was determined, the corresponding genomic region was extended by 5 kb on either side. Transcript sequences were then realigned on the extended region using EST_GENOME [96] (mismatch 2, gap penalty 3) to define transcript exons [97]. These transcript models were fused by a single linkage clustering approach, in which transcripts from the same genomic region sharing at least 100 bp are merged [98]. These clusters were used to detect alternative splicing events [99].

### Detection, functional annotation and comparative analysis of carbohydrate-active enzymes

Catalytic modules specific to carbohydrate-active enzymes (CAZymes: GHs, glycoside transferases, PLs and CEs) and their ancillary CBMs in fungi were searched by comparison with a library of modules derived from all entries of the Carbohydrate-Active enZymes (CAZy) database [73]. Each protein model was compared with a library of over 100,000 constitutive modules (catalytic modules, CBMs and other non-catalytic modules or domains of unknown function) using BLASTP. Models that returned an e-value passing the 0.1 threshold were automatically sorted and manually analyzed. The presence of the catalytic machinery was verified for distant relatives whenever known in the family. The models that displayed significant similarities were retained for functional annotation and classified in the appropriate classes and families.

Many of the sequence similarity-based families present in CAZy do not coincide with a single substrate or product specificity and, therefore, they are susceptible to grouping proteins with different Enzyme Commission (EC) numbers. Similarly to what has been provided for other genome annotation efforts, we aimed at producing annotations for each protein model that will survive experimental validation, avoiding over-interpretation. A strong similarity to an enzyme with a characterized activity allows annotation as 'candidate activity', but often for a safe prediction of substrate specificity, annotation such as 'candidate α- or β-glycosidase' may be provided, as the stereochemistry of the α- or β-glycosidic bond is more conserved than the nature of the sugar itself. Each protein model was compared to the manually curated CAZy database, and a functional annotation was assigned according to the relevance. All uncharacterized protein models were thus annotated as 'candidates' or 'related to' or 'distantly related to' their characterized match as a function of their similarity. The overall results of the annotation of the set of CAZymes from *P. anserina *were compared to the content and distribution of CAZymes in several fungal species (Danchin *et al*., in preparation) in order to identify singularities in the families' distributions and sizes per genome (data not shown). This allowed the identification of significant expansions and reductions of specific CAZyme families in *P. anserina*.

### Growth tests

M2 minimal medium contained per liter: 0.25 g KH_2_PO_4_, 0.3 g K_2_HPO_4_, 0.25 g MgSO_4_·7H_2_O, 0.5 g urea, 0.05 mg thiamine, 0.25 μg biotine and trace elements [100], 12.5 g agar; it was adjusted to pH 7 with KH_2_PO_4_. Standard M2 contains also 5.5 g/l dextrine, which was replaced by the other tested carbon sources. Sucrose, D-glucose, D-fructose, D-xylose, inulin, Apple pectin, carboxymethyl cellulose and Birchwood xylan were from Sigma-Aldrich (Gillingham, UK) and were added before autoclaving. *P. anserina *was grown for 7 days at 25°C.

## Abbreviations

BAC, bacterial artificial chromosome; CAZymes, carbohydrate-active enzymes; CBM, carbohydrate binding module; CDS, coding sequence; CE, carbohydrate esterase; EST, expressed sequence tag; GH, glycoside hydrolase; MSUD, meiotic silencing of unpaired DNA; PKS, polyketide synthase; PL, polysaccharide lyase; RIP, repeat induced point mutation.

## Authors' contributions

RD and PS initiated the project. Funding was secured thanks to Genoscope and CNRS. PS coordinated the project. AC, JMA, BS, JP, VA, PW and JW acquired and assembled the sequence. FM, EE, PS, ASC, AB, HK, EC, MDC, MP, VC, SA, AB and CHS contributed to the assembly and juxtaposition of the sequence with the genetic map. BPL, RD and CHS constructed the cDNA libraries. SG and OL developed the bioinformatic tools. MP and CDS analyzed the EST database. OJ and RD performed the synteny analysis. DG identified the non-coding RNA. EE, OL and PS analyzed the repeated sequences and the gain/loss of genes. EE, OL, FM, VBL, RPdV, EB, PMC, EGJD, BH, REK and PS analyzed the genome content. EE, OL, FM, CDS, PW, RPdV, PC, VB, AS, RD and PS contributed to writing the manuscript. All authors read and approved the final manuscript.

## Additional data files

The following additional data are available with the online version of this paper. Additional data file 1 is a figure of the *P. anserina *genome map as defined by classic genetic markers and molecular markers, mainly microsatellites, that are polymorphic between strains S and T. Additional data file 2 is a table listing CDSs potentially expressed through frame-shift and read-through. Additional data file 3 is a table listing transposons and transposon-like elements of the *P. anserina *genome. Additional data file 4 is a table listing segmental duplications in the *P. anserina *genome. Additional data file 5 is a table listing CDSs putatively involved in genome protection mechanisms. Additional data file 6 is a list of putative CDSs involved in (hemi-)cellulose and pectin degradation.

## Supplementary Material

Additional data file 1 The *P. anserina *genome map as defined by classic genetic markers and molecular markers, mainly microsatellites, that are polymorphic between strains S and T. Click here for file

Additional data file 2 CDSs potentially expressed through frame-shift and read-through. Click here for file

Additional data file 3 Transposons and transposon-like elements of the *P. anserina *genome. Click here for file

Additional data file 4 Segmental duplications in the *P. anserina *genome. Click here for file

Additional data file 5 CDSs putatively involved in genome protection mechanisms. Click here for file

Additional data file 6 CDSs involved in (hemi-)cellulose and pectin degradation. Click here for file
