# Supplementary material for: The genome sequence of the model ascomycete fungus Podospora anserina
Source: Genome Biol. 2008 May 6;9(5):R77. doi: 10.1186/gb-2008-9-5-r77 (PMC2441463; doi:10.1186/gb-2008-9-5-r77)
Supplement: Additional data file 1 — The P. anserina genome map as defined by classic genetic markers and molecular markers, mainly microsatellites, that are polymorphic between strains S and T. [file gb-2008-9-5-r77-S1.doc]

**5**

**6**

**7**

**1**

**2**

**3**

**4**

**1**

**4**

**5**

**6**

**8**

**9**

**10**

**2**

**3**

**7**

**2**

**4**

**1**

**3**

**1**

**3**

**2**

**1**

**5**

**2**

**3**

**4**

**6**

**2**

**3**

**1**

**1**

**2**

**3**

**rDNA**


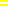


**1**

**2**

**4**

**3**

1 Mb

**hylaC1**

**1.1-13**

***IDC3***

***su3***

**8.1-1**

**8.1-3**

**5PH2**

**5PH1**

**5PH3**

***Patrk-2***

***PaMpk2***

***su8***

**IPH9**

**5PH6**

**IPH22**

***PaASK1*/5PH4**

***PaNox2***

**5PH5**

**IPH13**

**IPH24**

**IPH32**

**IPH38**

**IPH35**

**69cent**

***CatP1***

**214-10**

***cox5***

**709/384**

**108k**

**414k**

**684k**

**879k**

**1123k**

**1317k**

***pex7***

**IPH12**

**IPH19**

**109k**

**IPH27**

**214-6**

***PaTrx2***

**214-1**

**105-1**

**105-4**

**6PH4**

**6PH3**

**IPH36**

***mus-51***

**VIB-2**

**rana202**

**6disco1**

***HymA***

***cat2***

***scad***

***PaTLK2***

***mdm10***

***leu1-1***

**7G14**

**7G6**

***PaTrX3***

**7G12**

**7G13**

**7G11**

**7D4**

**7D6**

***PaMKK1***

**7G15**

**7G8**

**7G10**

***su4***

***polG***

***su2/AS2***

***Patrk-1***

**7D1**

**IPH34**

**IPH33**

**7G16**

**7G19**

***PaTrX1***

**7PH1**

**disco427**

***PaVTS1***

***PaNoA1***

***Pa_1_8470***

**1PH3**

***PaPls1***

**1.1-6**

**crapC1**

***PaMpk3***

***Papcn1***

***AS1***

***cytC1***

***PaNox3***

**IPH11**

**1G1**

**1G6**

**1PH2**

***PaNox1***

**1G8**

***car1***

**1G3**

***ABC1***

**1G11**

**1G4**

**1G12**

**1G5**

**inc64-1**

**inc64-2**

**1D20**

**1D22**

**1D24**

**1D25**

**1D10**

**1D11**

**1D12**

**1D14**

**1G16**

**1G17**

**1G19**

**1G20**

**1D9**

**1D6**

**1D2**

**1D1**

***fle1***

***pex5***

***AS4***

**1D28**

**1D29**

***rmp1***

***mat***

**1D34**

**1D33**

**1D32**

**IPH16**

**IPH15**

***mod-D***

***fox2***

**1D42**

**rein6**

**rein7**

**rein8**

***pex10***

**1PH4**

**1PH1**

**rein5**

***IDC2***

**1.1-3**

***mtHMG1***

***ami1***

***su12***

**disco42**

**RAINII1**

**2HetDav3**

***Het-D***

**II13**

**II23**

***mod-E***

**II11**

***mfp***

***PaPKS1***

***PaMpk1***

**IPH21**

**inc50.1-5**

**II30**

**II14**

**II29**

**II28**

**II27**

**IPH10**

**II12**

**II10**

**II33**

**II32**

**II24**

***pah1***

**II7**

**2HetDav1**

**II8**

***Tom70***

**inc50.1-4**

**II2**

**II20**

**II21**

**II22**

**II3**

**II4**

***oxa1***

**IPH23**

***PaMKK2***

**CENII1**

***PaNAT1***

**II1**

**Leti32**

**XA4**

***IDC1***

**inc133-2**

***ura5***

***PaAOX***

**het-S**

**249k**

**175k**

**IPH8**

**IPH7**

***idi-1***

**651k**

**409k**

**198k**

***Pania***

**539/770**

**440/651**

**inc133-1**

***idi-7***

**inc133-3**

***AS6***

**130k**

***cit1***

**415k**

***cro1***

***ABC2***

**397k**

**620k**

***pex12***

**B3-1**

**XA1**

**XA2**

**XA3**

**Leti21**

**Leti22**

**120K Leti23**

**Leti24**

**Leti25**

**Leti27**

**Leti28**

**Leti30**

**Leti26**

**Leti31**

**Leti33**

**B3-3**

**B3-4**

**B3-5**

***lys2***

***mei4***

***su1***

**am12**

**FM18**

***153***

**4am6**

**4am7**

**IPH18**

**4am14**

**4am13**

**4am5**

**4am2**

**4am1**

**FM24**

**4SNP1**

**4SNP2FM21**

**FM22**

**FM23**

**FM19**

**FM16**

**FM15**

**am10**

**am8**

**am11**

**IPH30**

**36crap**

***pex11***

***grisea***

***sir2***

***PaNAT2***

**7G17**

***P. anserina***

**Genome MAP**

**CapR1**

**mid26**

**mtDNA**

**SYL1**

**SYL2**

**SYL3**

**SYL5**

**SYL7**

**SYL10**

***echA***

***CatP2***

***Hom3***

***Bek1***

***Hom4***

**Classical genetic markers in red and molecular markers in black.**

**The double arrows indicate non-oriented supercontigs and the red dots the centromeres. The supercontigs are boxed and numbered.**
