# Supplementary material for: The genome sequence of the model ascomycete fungus Podospora anserina
Source: Genome Biol. 2008 May 6;9(5):R77. doi: 10.1186/gb-2008-9-5-r77 (PMC2441463; doi:10.1186/gb-2008-9-5-r77)
Supplement: Additional data file 2 — CDSs potentially expressed through frame-shift and read-through. [file gb-2008-9-5-r77-S2.doc]

| **Error** | **CDS** | **EST** | **Conservation in other fungi** | **Putative function** |
| --- | --- | --- | --- | --- |
| +1 frameshift | Pa_5_3030 | no |  | similar to *S. cerevisiae* HOL1 |
| -1 frameshift | Pa_1_8470 | yes | all pezizomycotina | similar to *S. cerevisiae* YIP3 |
|  | Pa_2_10750 | no |  | similar to *Aspergillus phoenicis* cpds precursor |
|  | Pa_5_740 | no |  | similar to *Cochliobolus carbonum* TOXA |
|  | Pa_1_16180 | no | present in many retrotransposons | gag-pol from retrotransposon YETI |
| read-through | Pa_1_16470 | no |  | similar to *N. crassa* LAC2 |
|  | Pa_3_1290 | yes |  | *P. anserina*-specific CDS of unknown function |
|  | Pa_5_770 | no |  | *P. anserina* specific CDS of unknown function |
|  | Pa_5_7410 | yes | *C. globosum* | *P. anserina* and *C. globosum* specific CDS of unknown function |
|  | Pa_5_12470 | no |  | dikaryomycota-specific CDS of unknown function |
|  | Pa_6_10530 | yes |  | dikaryomycota-specific CDS of unknown function |
|  | Pa_6_3310 | yes |  | *P. anserina*-specific CDS of unknown function |
|  | Pa_1_8040 | yes | some sordariomycetes | sordariomycete-specific CDS of unknown function |
|  | Pa_1_18100 | no |  | similar to *P. anserina* het-E |
