# Supplementary material for: The genome sequence of the model ascomycete fungus Podospora anserina
Source: Genome Biol. 2008 May 6;9(5):R77. doi: 10.1186/gb-2008-9-5-r77 (PMC2441463; doi:10.1186/gb-2008-9-5-r77)
Supplement: Additional data file 3 — Transposons and transposon-like elements of the P. anserina genome. [file gb-2008-9-5-r77-S3.doc]

| **Family** | **Name** | **Element size (bp)** | | **Estimated^1^**  **copy number** | |
| --- | --- | --- | --- | --- | --- |
|  |  |  |  |  |  |
| **DNA transposons** |  |  | **ITR size** |  |  |
| mutator-like | dendrobates | 3 449 | 65 | 3 | |
|  |  |  |  |  | |
| Tc1/mariner-like | discoglosse | 1 867 | 49 | 153 | |
|  | pelobate | 1 948 | 45 | 20 | |
|  | rainette | 1 298 | 41 | 9 | |
|  | scaphiopus | 1 428 | 23 | 1 | |
|  |  |  |  |  | |
| MITE | altiphrynoides | 403 | 50 | 5 | |
|  | discodeles | 257 | 40 | 49 | |
|  | mantella | 383 | 46 | 18 | |
|  |  |  |  |  |  |
| **LTR retrotransposons** |  | **full element** | **LTR** | **full element** | **solo LTR** |
| copia/Ty1 | centrolene | 5 918 | 182 | 18 | 14 |
|  | hyla | 6 466 | 630 | 10 | 63 |
|  | nephelobates | 5 594 | 445 | 7 | 0 |
|  |  |  |  |  |  |
| gypsy/Ty3 | crapaud | 7 372 | 400 | 80 | >300 |
|  | grenouille | 11 866 | 360 | 20 | 53 |
|  | pipa | >4 242 | ? | 1 | 0 |
|  | yeti | 6 943 | 355 | 2 | 37 |
|  |  |  |  |  |  |
| solo LTR | boophis | ? | 367 | 0 | 3 |
|  | alytes | ? | 230 | 0 | 3 |
|  | arthroleptis | ? | 238 | 0 | 10 |
|  | ascaphus | ? | 170 | 0 | 6 |
|  | astylosternus | ? | 172 | 0 | 12 |
|  | atelopus | ? | 601 | 0 | 9 |
|  | bufoides | ? | 153 | 0 | 6 |
|  | capensibufo | ? | 256 | 0 | 11 |
|  | cochranella | ? | 155 | 0 | 18 |
|  | crepidophryne | ? | 137 | 0 | 6 |
|  | cyclorana | ? | 194 | 0 | 8 |
|  | dendrophryniscus | ? | 73 | 0 | 5 |
|  | eleutherodactylus | ? | 58 | 0 | 14 |
|  | nyctibates | ? | 169 | 0 | 5 |
|  | pelodytes | ? | 128 | 0 | 8 |
|  | rana | ? | 508 | 0 | 17 |
|  | xenopus | ? | 235 | 0 | 16 |
|  |  |  |  |  |  |
| **helitron** |  |  |  |  |  |
|  | epipadobates | 8388 | | 22 | |
|  |  |  |  |  |  |
| **unknown affinity** |  |  |  |  |  |
|  | adenomus | 382 | | 14 | |
|  | allophryne | 268 | | 9 | |
|  | amolops | 585 | | 31 | |
|  | andinophryne | 122 | | 12 | |
|  | ansonia | 90 | | 5 | |
|  | batrachyla | 192 | | 20 | |
|  | bombina | 680 | | 10 | |
|  | bombinator | 472 | | 5 | |
|  | brachycephalus | 259 | | 5 | |
|  | bufo | 699 | | 9 | |
|  | callulops | 302 | | 5 | |
|  | cardioglossa | 182 | | 5 | |
|  | churamiti | 143 | | 6 | |
|  | lechriodus | 246 | | 13 | |
|  | leptodactylodon | 570 | | 22 | |
|  | leptopelis | 112 | | 6 | |
|  | microhyla | 1179 | | 8 | |
|  | platymantis | 593 | | 8 | |
|  | schoutedenella | 241 | | 7 | |

^1^Estimations are based on the number of copies found in the present draft version of the genome. In subsequent assembly, numbers may decrease if separate copies are combined, or increase if new copies are found in sequence gaps.

Transposons were manually annotated by comparing with known fungal transposons using BLAST. Potentially active elements were the yeti retrotransposon and rainette DNA transposon, although no transposition of these elements has been detected so far. One copy of yeti located on chr 1 has an open reading frame containing a single -1 frameshift site with the correct context for being skipped during translation as observed for other elements of this class. All the other yeti copies are defective. Rainette is present in nine copies, four of which carry transposase CDS devoid of non-sense mutation. Two copies are strictly identical, while the seven others are over 99% identical. None of the other transposons display such a high level of identity, suggesting that rainette is the most recent invader of *P. anserina* genome. Examination of other *P. anserina* geographic isolates (data not shown) shows that this element is present in most of the strains although not in all positions detected by the present sequence in the S strain, but absent from the evolutionarily distant T strain (this strain is often considered as a sibling species of *P. anserina* called *P. comata*). This suggests that rainette arrived in the S strain and its relatives after their divergence from the T strain, and the patchy repartition of rainette suggests that it may still be active.

In addition to repeated sequences that clearly originate from transposons, 19 additional repeated sequences are dispersed within the genome in copy numbers higher than 5 and up to 31, and do not have any obvious coding capacity (unknown affinity sequences). Their origin is at present obscure; however the fact that they are present at copy numbers higher than 5 suggests that most of these sequences may amplify through a specific mechanism, some possibly being degenerate transposons. Alternatively, they may code for functional RNA genes whose sequences were not sufficiently conserved during evolution to be recognized by homology search.
