# Supplementary material for: The genome sequence of the model ascomycete fungus Podospora anserina
Source: Genome Biol. 2008 May 6;9(5):R77. doi: 10.1186/gb-2008-9-5-r77 (PMC2441463; doi:10.1186/gb-2008-9-5-r77)
Supplement: Additional data file 4 — Segmental duplications in the P. anserina genome. [file gb-2008-9-5-r77-S4.doc]

| size (bp) | number of Segmental Duplications |
| --- | --- |
| 60-500 | 209 |
| 500-1 000 | 35 |
| 1 000- 2000 | 29 |
| 2 000-10 000 | 23 |
| 10 000-20 000 | 3 |

Segmental duplications were manually annotated by searching similar sequence by BLASTN to avoid detection of ancient duplications. 262 are duplicated sequences, 26 are triplicated sequences, 8 are quadriplicated sequences, 2 are pentaplicated sequences and 1 set contains 13 copies. The pentaplicated sequences code for the U1 (four potential genes and one pseudogene) and U6 snRNA genes. The 13-member set is composed of genes and pseudogenes of the het family [65]. In most instances, one member of the other segmental amplifications covers a CDS (or coding RNA), indicating that they originate from *bona fide* amplification of a coding portion of the *P. anserina* genome.
