# Supplementary material for: The genome sequence of the model ascomycete fungus Podospora anserina
Source: Genome Biol. 2008 May 6;9(5):R77. doi: 10.1186/gb-2008-9-5-r77 (PMC2441463; doi:10.1186/gb-2008-9-5-r77)
Supplement: Additional data file 5 — CDSs putatively involved in genome protection mechanisms. [file gb-2008-9-5-r77-S5.doc]

|  |  | *P. anserina^1^* | *N. crassa* |
| --- | --- | --- | --- |
| **RIP** |  |  |  |
| RID (*N. crassa*)/  Masc1(*A. immersus*) | Putative DMT, essential for RIP [59] and for Methylation Induced Premeotically or MIP [132] | Pa_1_19440 | NCU02034.3 |
| Dim-5 (*N. crassa*) | H3 3mK9 HMT essential for RIP [133] also known as SUVH4 in *Arabidopsis thaliana* [134, 135] | Pa_6_990 | NCU04402.3 |
|  |  |  |  |
| **Quelling** |  |  |  |
| QDE-1 (*N. crassa*) | RdRP, essential for quelling [136] | Pa_7_4790 | NCU07534.3 |
| QDE-2 (*N. crassa*) | Argonaute-like protein, essential for quelling [137] | Pa_4_8620 | NCU04730.3 |
| QDE-3 (*N. crassa*) | RecQ helicase, essential for quelling [138] | Pa_1_10750 | NCU08598.3 |
| DCL1 (*N. crassa*) | Dicer-like protein, involved in quelling [139] | Pa_6_6150 | NCU08270.3 |
| DCL2 (*N. crassa*) | Dicer-like protein, involved in quelling [139] | Pa_3_7970 | NCU06766.3 |
| QIP (*N. crassa*) | Putative exonuclease protein, involved in quelling [140] | Pa_7_9210 | NCU06766.3 |
|  |  |  |  |
| **MSUD** |  |  |  |
| SAD-1 (*N. crassa*) | RdRP essential for MSUD [48] | Pa_1_19900 | NCU02178.3 |
| SAD-2 (*N. crassa*) | Essential for MSUD [141] | Pa_2_9830 | NCU04294.3 |
|  |  |  |  |
|  |  |  |  |
| **DNA methylation** |  |  |  |
| Dim-2 (*N. crassa*)  Masc2 (*A. immersus*)  MET1 (*A. thaliana*)  Dnmt1 (*M. musculus*) | *De novo* CpN DMT [58]  DMT [142]  Maintenance CpG DMT [143]  De novo/maintenance CpG DMT [144] | Pa_5_9100 | NCU02247.3 |
| CMT3 (*A. thaliana*) | Maintenance CpNpG DMT with chromodomain [145, 146] | None | None |
| DRM2 (*A. thaliana*) | *De novo* CpN DMT [147] | None | None |
| Dnmt3a (*M. musculus*) | *De novo* CpG DMT [148] | None | None |
| Dnmt3b (*M. musculus*) | *De novo* CpG DMT [148] | None | None |
| HP1 (*N. crassa*) | Heterochromatin factor, essential for CpN methylation [60] | Pa_4_7200 | NCU04017.3 |
|  |  |  |  |
| **Chromatin remodelling factors** |  |  |  |
| HDA6 (*A. thaliana*) | Histone deacetylase involved in CpG methylation [149, 150] | Pa_1_5330 | NCU00824.3 |
| HDT1 (*A. thaliana*) | Histone deacetylase involved in CpG methylation [151] | None | None |
| SIR2 (*S. cerevisiae*) | NAD-dependant histone deacetylase involved in TGS [152] | Pa_4_8570 | NCU04737.3 |
| DDM1 (*A. thaliana*) | SWI2/SNF2-like protein involved in CpN methylation [153, 154] | Pa_4_2720 | NCU03875.3 |

DMT: DNA Methylase, HMT: Histone Methyl Transferase, RdRP: RNA dependent RNA polymerase.

^1^ Orthologues of the genes involved in gene silencing and chromatin remodelling were found by the BRH method. Based on their sequence, all the proteins seem functional. Some have corresponding ESTs.
